# Implementing PROMs where care is complex: lessons from trauma-focused mental health services for refugees

**DOI:** 10.3389/fpsyt.2026.1770884

**Published:** 2026-03-19

**Authors:** Stine Bjerrum Moeller, Linda Nordin, Viktoria Bernicken, Lotte Kring

**Affiliations:** 1 Department of Multidisciplinary Trauma Treatment, Mental Health Services, Region of Southern Denmark, Middelfart, Denmark; 2 Department of Psychology, University of Southern Denmark, Danish Center of Psychotraumatology, Odense, Denmark; 3 DIGNITY- Danish Institute Against Torture, Copenhagen, Denmark; 4 Department of Psychology, Lund University, Lund, Sweden

**Keywords:** implementation, mixed method analysis, patient reported clinical outcomes, routine outcome assessment, trauma-affected refugees, reflexive theamatic analysis

## Abstract

**Background:**

Trauma-focused psychological interventions are central to treating PTSD and CPTSD among trauma-affected refugees, yet it remains unclear how patient-reported outcome measures (PROMs) can be meaningfully implemented in high-complexity clinical settings. This study examined real-world use of a web-based PROM system embedded in routine care for trauma-affected refugees in Denmark.

**Methods:**

We conducted a convergent, embedded mixed-methods case study at a specialist outpatient clinic participating in the Danish Trauma Database for Refugees (DTD). Quantitative data comprised (a) system-generated flow data for all patients referred between February 2023 and August 2025 (N = 634), describing registration, consent, and assessment completion at baseline, end of treatment, and 6-month follow-up, and (b) a clinician survey on usability and clinical value (n = 15). Qualitative data consisted of 10 semi-structured interviews with two clinician–patient dyads conducted at baseline, mid-treatment, and post-treatment, analyzed using reflexive thematic analysis. Findings were integrated across data sources to address implementation, perceived usability, experiences of use, and how these perspectives intersect.

**Results:**

Of 634 registered patients, 270 (42%) provided active research consent. Baseline PROM completion was moderate, with most patients contributing at least partial data, whereas completion declined substantially at post-treatment and follow-up (e.g., 77% and 90% of assessments unregistered, respectively). Clinicians rated technical usability as acceptable but reported limited perceived impact on clinical insight, personalized care, and interdisciplinary collaboration; half expressed concern that research demands risked overshadowing clinical priorities. Qualitative analyses identified three overarching themes: (1) the therapeutic relationship as the primary outcome, with PROMs secondary to being heard and recognized; (2) PROMs as routinised yet relationally negotiated tools, used mainly at intake; and (3) ongoing tension between standardization and flexibility as clinicians adapted PROM use to patients’ capacities and perceived vulnerability.

**Conclusion:**

In this trauma-focused refugee service, PROMs were only partially implemented and mainly used at baseline. Barriers were primarily epistemic and ethical rather than technical, reflecting concerns about clinical relevance, workflow fit, and protection of “vulnerable” patients. Sustainable PROM implementation in such settings likely requires co-created, reflexive approaches that prioritize epistemic fit, relational care, and proportionate inclusion over procedural compliance.

## 1 Introduction

Trauma-focused psychological interventions are central to treating PTSD and CPTSD among trauma-affected refugees (1). Patient-reported outcome measures (PROMs), standardized questionnaires completed by patients about their health and functioning, offer a structured way to integrate the patient’s voice into care, supporting shared decision-making, communication, and treatment tailoring (2, 3). This makes PROMs a potentially relevant strategy for improving treatment effects in general, and particularly in relation to complex disorders. As discussed by Basch (4), systematic use of PROMs can enhance the quality and safety of care by enabling timely detection of problems, improving symptom management, and fostering more responsive and person-centered treatment, an approach particularly relevant for complex disorders.

Cochrane reviews indicate that routine PROM collection alone does not consistently improve mental-health outcomes, whereas providing timely, interpretable, and action-linked feedback can enhance detection and clinician–patient communication, with modest, context-dependent effects on outcomes (5, 6). To realize this potential, PROM results need to be summarized clearly and integrated into clinical encounters, so they are immediately actionable (5, 7). Nevertheless, implementation studies highlight persistent barriers, including uncertainty about clinical utility, limited training, and concerns about burden and workflow misfit, that can impede routine use (3, 8, 9).

### 1.1 PROMs in relation to refugee populations

Despite growing evidence of the value of PROMs in mental health care, little is known about how they can be implemented or perceived as useful in routine clinical settings for underrepresented and diverse populations, particularly refugees with complex psychiatric disorders such as PTSD and CPTSD (10). Existing work on PROMs in refugee settings has concentrated primarily on linguistic fit and psychometric performance across languages and cultures, with little attention to whether and how PROM use improves care. Reviews and empirical studies have mapped reliability/validity, translation practices, and cross-group measurement invariance, showing, for example, acceptable internal consistency for several PTSD measures but uneven evidence on responsiveness and measurement error in displaced populations, and stronger cross-group invariance for the HTQ than for the HSCL-25 (with cautions for between-group comparisons and change scores) (11–14). By contrast, there is limited evidence on implementation questions central to clinical services: when and how PROMs should guide decisions, how feedback structures clinician–patient communication, how the patient perspective is incorporated into goal setting, and which organizational conditions enable routine use in interpreter-mediated, high-complexity care. These gaps motivate the present study’s focus on real-world use, asking whether PROMs, when embedded in routine refugee mental-health services, are perceived to assist decision-making and to support communication while preserving the patient perspective.

### 1.2 Summary of implementation of PROMs in the study setting

In 2021, the Department of Multidisciplinary Trauma Treatment in the South Region of Denmark, joined the national collaboration behind the Danish Trauma Database for Refugees (DTD) (15), and, after preparatory work, began routine monitoring in 2023 at intake, end-of treatment, and at follow up. Guided by evidence on PROMs and implementation science, we introduced a web-based PROM system as a clinical tool to standardize diagnostic assessment and outcome assessment broader to support communication, shared decision-making, and care planning in routine treatment for trauma-affected refugees. Our aim was to embed a common, comparable language for symptoms and functioning in everyday practice so that patients’ reports could inform clinical formulation at intake and anchor a shared evaluation at the end of treatment. Rather than imposing fixed dashboards or scripts, we co-developed use with clinicians. Through working sessions and iterative feedback, we created a structured template for clinical conferences (intake and discharge) that positioned PROM discussion where it naturally fit the workflow, early for case formulation and again for treatment review. The template clarified responsibilities (who brings and summarizes the data) and used a small set of anchor questions (e.g., what the PROM profile adds to formulation; which patient-reported problems should shape goals and treatment approach; whether scores indicate red flags or need for referral/adjustment in treatment plan). We relied on simple baseline and end of treatment reports, leaving room for clinical judgement and local adaptation. Our preparation training-phase study (16) showed that experiential practice (brief pilots, role-plays, hands-on interpretation) built confidence, yet uptake depended on workflow fit and perceived clinical payoff. Clinicians felt more engaged when results clearly informed formulation and helped prioritize goals; enthusiasm waned when PROM tasks added time, duplicated documentation, or were hard to act on in interpreter-mediated sessions. Clinicians also frequently framed patients as “vulnerable,” adopting a protective stance that could delay or limit PROM use, although positive engagements were evident when PROMs supported structured assessment. To align with our context of difficult-to-treat psychiatric disorders (PTSD/CPTSD, comorbidity, social stressors), we emphasized alliance, pacing, and safety; offered optional, paced administration with permission to defer; enabled brief narrative notes alongside scores; simplified language; provided translated explanations and interpreter-friendly workflows (including an e-learning for interpreters); focused the core set on brief, validated measures of key clinical presentations and trauma history, alongside personal-recovery elements; allowed pen-and-paper completion with subsequent entry; and engaged a student assistant to monitor data flow, reconcile records, and prompt clinicians about missing data. Where needed, we supported staggered completion and encouraged clinicians to “review what we have” when data were partial. Our working assumption was that PROMs could add value in a difficult-to-treat population by securing systematic assessment and preserving the patient perspective in planning, even as early buy-in remained uneven.

### 1.3 The current study

Building on these preparatory phases, the present study moves from planning and preparing to examining real-world implementation and use of the PROM system within routine workflow (administration at baseline/intake, end of treatment and 6 months follow-up). Rather than enforcing a rigid protocol, we investigate implementation as it unfolded to understand how clinicians and patients made sense of and found value in using PROMs within routine care. We adopted an open, exploratory stance to clinicians’ and patients’ experiences to understand use in context.

Research questions:

1. To what extent is the PROM database implemented and used in routine mental health care for refugees?
2. How do clinicians perceive the usability and clinical value of the PROM database?
3. How do patients and clinicians experience the use of PROMs in supporting communication and care decisions?
4. How do insights from system-use data, clinician surveys, and dyadic interviews complement each other in understanding the use and impact of the PROM database?

## 2 Material and methods

### 2.1 Study setting and participants

We conducted a convergent, embedded mixed-methods case study at the Department of Multidisciplinary Trauma Treatment, Region of Southern Denmark, a specialist outpatient service that treats approximately 300 trauma-affected refugees annually. The study examined the implementation of the existing Danish Trauma Database for Refugees (DTD), a national collaboration that collects routine patient-reported outcomes and sociodemographic data across five outpatient clinics to support both clinical care and research (15). The department joined the DTD in 2021 and, following staff training and pilot workflow testing, started routine monitoring in February 2023.

Patients are referred to the department by hospital units, general practitioners, or private psychiatrists for assessment and treatment of trauma-related mental health problems. Individuals are offered treatment if they meet clinic eligibility criteria; those who do not are redirected to appropriate primary-care or other specialist services in coordination with the referring provider. In line with national policy, only refugees who have been granted asylum are eligible for treatment.

Within the DTD, PROM assessments are scheduled at pre-treatment (baseline), post-treatment, and 6-month follow-up. The core battery includes sociodemographic variables and validated measures of trauma exposure and traumatic brain injury, PTSD/CPTSD, anxiety/depression, pain, disability/functioning, personal recovery, and post-migration stressors: the International Trauma Exposure Measure (ITEM) (17) and HTQ-Part 3 (head injury) (18); ICD-11 PTSD/CPTSD via the International Trauma Questionnaire (ITQ) (19) and the clinician-administered International Trauma Interview (ITI) (20); disability/functioning via the 12-item WHO-DAS (21); anxiety/depression via the HSCL-10 (22); pain severity and interference via the short Brief Pain Inventory (BPI-SF) (23); personal recovery via Brief INSPIRE-O (24, 25); and post-migration living difficulties via a nine-item version of the post-migration living difficulties (PMLD) checklist (26). Instruments have been selected for cross-cultural use and service feasibility; further psychometric and scoring details are provided in the DTD protocol (15).

### 2.2 Legal and ethical approval

The DTD-collaboration is governed by a formal contract regulation data security and integrity, ethical approval, data access, transfer, and storage (15). The current study’s data collection was based on written informed consent and stored according to the hospital´s ethics approval system license number OP_2288. We stored data in the OPEN (Open Patient data Explorative Network, Odense University Hospital, Region of Southern Denmark).

### 2.3 Sources of evaluation

This mixed-methods study was based on both quantitative and qualitative sources of data. Quantitative data comprised two complementary sources. First, we extracted system-generated flow data from the PROM database, capturing patient monitoring activities over time, including registration, consent status, and completion rates of baseline, end of treatment, and follow-up assessments. These data provided a detailed overview of how the database was implemented and used across the care pathway. Second, we conducted a clinician survey assessing clinicians’ perceptions of the DTD monitoring system’s usability, clinical value, and integration into everyday practice. Together, these quantitative sources offered a broad perspective on both the operational reach of the system and its perceived utility among clinicians.

To complement and deepen these insights, qualitative data were collected through interviews with two clinician–patient dyads, encompassing two patients and their respective therapists (four informants in total). The qualitative component followed a case study approach designed to capture depth and nuance rather than data saturation, consistent with qualitative research traditions. For each dyad, we conducted a series of semi-structured individual interviews at key points in the treatment trajectory: baseline (treatment initiation), mid-treatment, and post-treatment. In one dyad, the patient ended treatment earlier than anticipated; thus, only baseline and post-treatment interviews were completed. A total of 10 interviews were conducted, each lasting approximately one hour (range: 53–76 minutes), with one shorter interview of 35 minutes conducted by telephone due to feasibility constraints.

Interview guides were collaboratively developed by the research team (SBM, VLB, LK) without reliance on a specific theoretical framework, ensuring pragmatic alignment with the overarching research questions and clinical context. The same guide was used for both dyads at each stage, while separate guides were designed for each phase to capture stage-specific dynamics while maintaining consistency.

As shown in **Table 1**, the interview guides addressed complementary perspectives across treatment stages. Clinician interviews emphasized the role of DTD data in assessment, planning, and evaluation, while patient interviews focused on expectations, lived experiences of treatment, and perceptions of progress and outcomes. Together, these perspectives provided a multifaceted view of how DTD was integrated into clinical practice and experienced by both patients and clinicians.

**Table 1:** Summary of clinician and patient interview guides at baseline, mid-treatment, and post-treatment.

Stage	Clinician interviews	Patient interviews
Baseline	Focused on the use of DTD assessments, patient responses, technology use, and how DTD data informed clinical understanding, treatment planning, and goal setting.	Focused on patients’ background and reasons for seeking help, expectations for treatment, experiences of the assessment process, technology use, and hopes and goals for therapy.
Mid-treatment	Focused on the status of the treatment, how treatment was planned and adjusted, the role of DTD data, and the setting and revisiting of treatment goals collaboratively with the patient.	Focused on patients’ experiences of ongoing treatment, perceived progress, the role of assessments and DTD data in their care, and whether their hopes or goals had shifted since baseline.
Post-treatment	Focused on clinicians’ evaluation of the treatment process and outcomes, the use of DTD data in treatment planning and decision-making, and reflections on the potential for future use of DTD.	Focused on patients’ reflections on the treatment received, changes in symptoms and daily life, how expectations and goals were met, and their views on the usefulness of assessments and DTD data throughout treatment.

The interviews were conducted by two researchers: a psychology master’s student completing an internship at the department (VLB), and a PhD fellow in psychology with a background in anthropology (LK). LK had previously been employed at the department and involved in the implementation of the DTD database. Both interviewers were familiar with the department, its patient population, and the overall project, but had only limited knowledge of the two clinicians and none with the patients. VLB conducted all patient interviews, while LK conducted all clinician interviews.

This embedded mixed-methods approach allowed individual case experiences to be interpreted within the broader context of usage patterns and clinician perspectives, thereby combining breadth and depth. The integration of quantitative and qualitative findings during analysis enabled a richer, more nuanced understanding of the PROM database’s role in clinical care.

### 2.4 Data analysis

Quantitative data were analyzed descriptively to provide an overview of implementation fidelity, including database usage patterns and assessment completion rates, as well as clinician-reported usability of the system. Qualitative data were analyzed using reflexive thematic analysis (RTA) to explore patterns of meaning across interviews (27, 28). All interviews were transcribed verbatim by VLB, a process that also contributed to analytic familiarization. The analysis followed Braun and Clarke’s six recursive phases of reflexive thematic analysis (27, 28), approached as a dynamic and iterative process rather than a linear sequence. Movement back and forth between phases was central to refining codes, reworking themes, and deepening engagement with the data. The analytic process involved: (1) familiarization with the data through repeated reading and initial note-taking, (2) generating initial codes for each interview at the level of each dyad, (3) developing preliminary themes across dyads based on patterns identified within and between dyads, (4) reviewing and refining these themes iteratively, attending to both convergence and divergence between patient and clinician perspectives, (5) defining and naming final themes across dyads, and (6) producing the analysis. The analysis was structured in two layers: first at the level of each dyad to capture within-dyad dynamics, and then across dyads to identify overarching patterns, shared experiences, and points of difference. This comparative layer enabled examination of patient and clinician perspectives both individually and relationally, situating each within the broader context of how PROM data were used and experienced in trauma-focused care.

## 3 Quantitative results and qualitative analyses

### 3.1 Implementation and utilization of the PROM database (research question 1)

**Figure 1:**
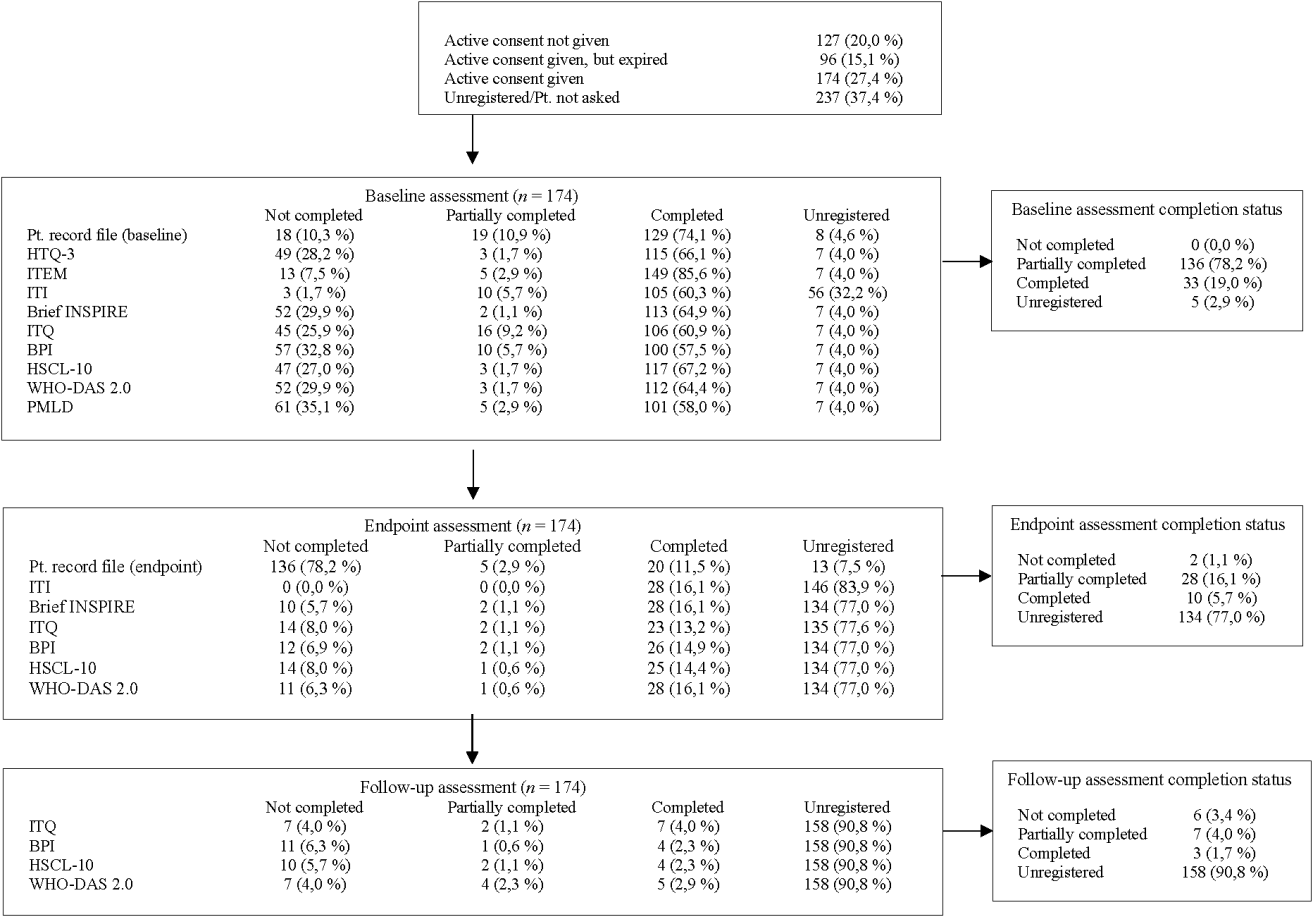
Flowchart of patients registered between 05 February 2023 and 04 August 2025 (*N* = 634).

To examine the extent to which the web-based PROM database was implemented and used in routine refugee mental health care, we analyzed system-generated flow data from all patients referred to the Department of Multidisciplinary Trauma Treatment between 5 February 2023 and 4 August 2025 (see **Figure 1**). During this period, a total of 634 patients were registered in the system. Of these, 270 (42%) provided active consent for their data to be used, 127 (20%) declined to provide active consent, and for 237 (37%) no consent status was registered (e.g., patients were not asked, or data not registered).

Among patients with active consent, baseline PROM data were collected as part of the intake process. Completion rates varied across measures, but most patients provided at least partial data. For example, the baseline patient record was completed in 74% of cases, while the majority of standardized instruments such as the HTQ-3, ITEM, Brief INSPIRE-O, and WHO-DAS II were completed around 60% of cases. Completion rates for the International Trauma Interview (ITI) were 60%, and for the trauma exposure measure, ITEM it was 85%. In contrast, completion rates dropped considerably at later assessment points. At end of treatment, 16% of patients had partially completed assessments and only 6% had complete data, while 77% of end of treatment assessments remained unregistered. Follow-up assessments showed even lower engagement, with 90% unregistered and only 2% fully completed.

### 3.2 Clinician perceptions of the PROM database: survey response profile (research question 2)

The survey was distributed to 20 clinicians. Of these, 12 (60%) completed all items, 3 (15%) provided partial responses, and 4 (20%) did not respond. One clinician (5%) did not provide research consent and was excluded from analysis, yielding an analytic sample of n = 15 (12 complete, 3 partially complete. Item-level denominators therefore vary and are reported per row in the figures.

**Figure 2:**
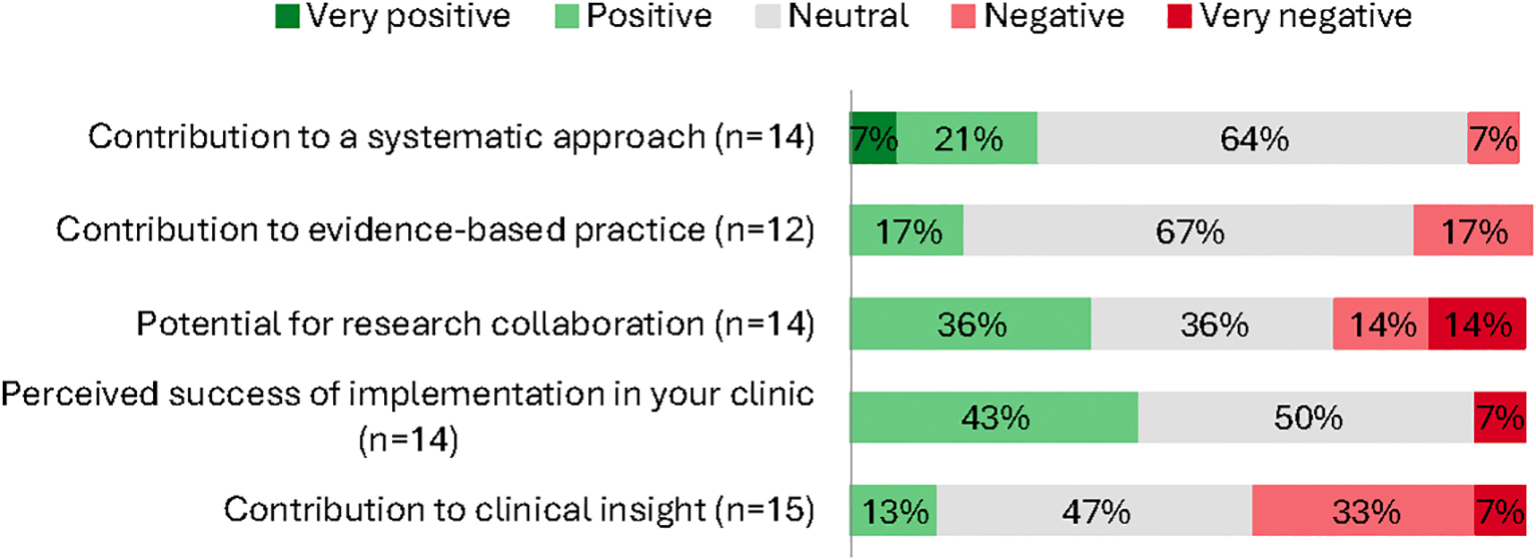
Clinician survey: proportional responses (very positive → very negative) by item (%; n varies).

Almost half of clinicians rated the implementation as successful (43% positive), while about one third indicated the database helped support a more systematic clinical approach (28% positive). In contrast, perceived contributions to clinical insight (13% positive) and evidence-based care (17% positive) were lower. Although the initiative was implemented nationally across five clinics, only 36% rated the research collaboration potential positively (**Figure 2**).

**Figure 3:**
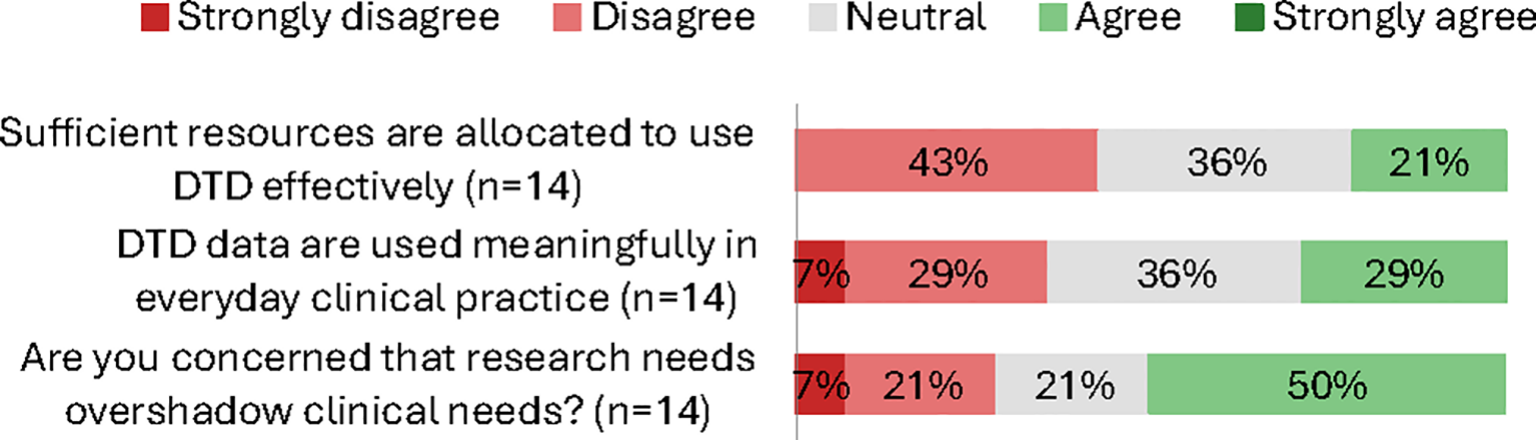
Clinician survey: proportional responses (strongly disagree → strongly agree) by item (%; n varies).

**Figure 4:**
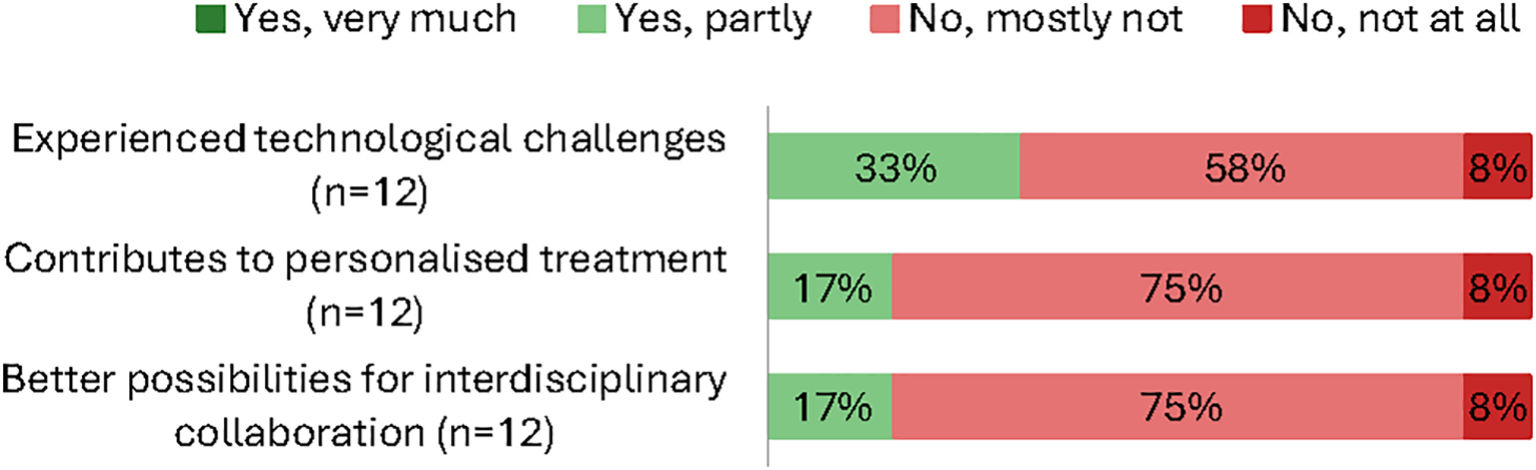
Clinician survey: proportional responses (yes, very much → no, not at all) by item (%; n varies).

Only about one third of clinicians reported that DTD data are used meaningfully in everyday practice (29% agree), and roughly one in five felt there were sufficient resources for effective use (21% agree). Half expressed concern that research needs may overshadow clinical needs (50% agree) (**Figure 3**). Almost two thirds reported none or only few technological problems using the database (≈67%). By contrast, only about one in five clinicians (17%) indicated that the database contributed to interdisciplinary collaboration or personalized care for the individual patient (**Figure 4**).

### 3.3 Patient and clinician experiences of PROM-supported care (research question 3)

Through the qualitative analytical process, three overarching themes were developed, capturing how patients and clinicians experienced and made sense of using PROMs in trauma-focused care: (1) Therapeutic relationship as a central outcome, (2) The everyday practice of assessment in psychiatric care, and (3) The tension between standardization and flexibility. Theme 2 is further divided into three interrelated subthemes, reflecting how assessments are practiced, adapted, and negotiated in everyday clinical contexts. Each theme is presented with interpretive analysis and illustrative quotations, highlighting both convergences and divergences between patient and clinician perspectives, and concludes with an analytic summary.

Pseudonyms are used for all participants. The pseudonyms assigned to clinicians are gender neutral. All identifying details have been changed to protect anonymity. These changes do not affect the substance of the analysis.

#### 3.3.1 Therapeutic relationship as a central outcome

##### 3.3.1.1 Dyad: Rami and Sam

One dyad consists of Rami, a man in his forties who fled a Middle Eastern country in 2015 and now lives in Denmark with his wife and children, and Sam, the psychologist working with him at the clinic, where Rami is currently enrolled for the second time. In the present course of treatment, he does not articulate any specific expectations but rather expresses a fundamental need to be heard and to share his experiences and emotions with someone willing to listen. This emphasis on being listened to reflects how his previous encounters with mental health services have shaped his current expectations of therapy:

*“Based on the experience I have from the treatments I’ve had before … I don’t really have any particular expectations, or I haven’t really thought about what I expect from ATT itself. But for me, in general, it’s about having someone who listens to me. [ … ] I feel that just sitting here and sharing this with someone who listens to me and understands me – that in itself helps me.”*

For Rami, the therapeutic relationship offers relief, containment, and recognition. The value of therapy lies less in specific tools and more in the opportunity to externalize distress; otherwise, he explains, the emotional burden spills into family life, in particular with his wife.

Sam, Rami’s therapist, describes expectations for the treatment as both hopeful and realistic:

*“Of course, you have to give hope and all that, right? But it also has to be realistic, right? And it has to be adjustable along the way, to see what fits him, you know?”*

Sam continues:

*“You just hope that it helps him to talk about these traumas, and that it might give him fewer nightmares and so on.”*

According to Sam, there has not been an explicit conversation devoted to aligning expectations with the patient. Following the assessment phase, both agreed to proceed with trauma-focused treatment, although the specific content and structure of the therapy had not yet been defined. Once the treatment has been fully explained, the patient will have the opportunity to reconsider or decline participation, Sam explains. This illustrates how care decisions are made progressively and paced according to what the therapist considers appropriate for the patient at a particular time. Sam emphasizes the importance of not overwhelming the patient with too much information at once, thereby ensuring engagement and understanding, and maintaining the possibility for the patient to opt out if needed. Sam also reflects on the broader challenge of involving patients in treatment decisions. Some patients, like Rami, do not always hold a clear opinion or preference and instead prefer the clinician, as the professional, to take the lead. In such cases, Sam presents different options for the patient to consider, while maintaining flexibility to revise or adapt the plan as therapy unfolds. In Rami’s case, Sam proposed a treatment approach informed both by the patient’s current situation – having previously received treatment without trauma-focused therapy – and by professional experience and preferred methods. However, Rami eventually ended therapy midway for several reasons, including the relocation of the clinic, which made travel too difficult. In the post-treatment interview, he also expressed reluctance to engage in trauma-focused therapy, stating that talking about past traumas was not perceived as helpful. Sam respected this decision, noting that therapy must be a shared responsibility:

*“It’s not me who should carry it all. He has to want it too … and I felt I was starting to overextend myself.”*

This dyad illustrates how collaborative ideals are continuously negotiated in practice, between respecting patient autonomy and exercising clinical judgment, between shared responsibility and professional boundaries, and between the therapeutic aim of empowerment and the realities of patient dependency within care relationships.

##### 3.3.1.2 Dyad: Anwaar and Robin

The other dyad includes Anwaar, a man in his fifties who fled from a country in the Middle East and now lives alone in Denmark, and Robin, the therapist working with him at the clinic. Anwaar has previously received treatment at the clinic on two occasions. Anwaar’s expectations for the current course of treatment are modest. He primarily seeks someone to talk to, to feel listened to, and to experience a sense of relief. His main hope is to replace some of his many negative thoughts and perspectives on life with more positive ones:

*“But I came here to feel that there is a background, that there is somebody can talk to you and make you feel better. [ … ] Maybe to increase the positive idea in my head and to lower the negative idea.”*

As with Rami, Anwaar’s expectations for the current treatment may be shaped by earlier therapeutic experiences, reflecting a trajectory of limited benefit from prior interventions and an accumulation of past experiences within mental health care. At the mid-treatment interview, Anwaar introduced new perspectives on the therapeutic process. Rather than focusing solely on his initial motivation for entering treatment, he now described therapy as a process *“to take some experience, to take some knowledge, to take some information”* from the therapist – resources he could then apply in everyday life. He framed therapy as a learning and exchange process in which new coping strategies emerge through the interaction between his own efforts and the therapist’s expertise. In this way, he understood treatment as collaborative, while also emphasizing his own active role and responsibility in using and carrying forward what he learned:

*“It’s together. [ … ] It’s between me and [the therapist].”*

Anwaar’s reflections highlight the layered character of collaboration in therapy. While viewing treatment as a joint process, he also stressed his responsibility for applying insights gained, while relying on the therapist’s professional judgment to determine suitable methods and structure the therapeutic course. This interplay between collaboration, patient agency, and professional authority shapes how therapy is both understood and enacted.

This perspective aligns with Robin’s approach. Robin describes treatment planning as a gradual process in which patient preferences develop over time. Echoing Sam, Robin portrays decision-making as relational, guided, and adaptive rather than fixed at the outset.

##### 3.3.1.3 Analytical summary

Across the dyads, therapy appears as a relationally guided process, where dialogue, trust, and the sense of being met as a person constitute central therapeutic mechanisms. PROMs and assessment data formed part of this process but were secondary to the relational encounter.

#### 3.3.2 The everyday practice of assessment in psychiatric care

Situated as the second one of the three overarching themes, this theme explores how PROMs are enacted and negotiated in everyday psychiatric care. It unfolds through three interrelated subthemes that together illustrate how assessments, although standardized and routine, are continuously adapted, shaped by relational dynamics, and at times set aside in practice.

##### 3.3.2.1 Routine, expected, and unremarkable

In both dyads, the patients perceived the assessment as an expected and routine part of entering treatment. Both patients were aware that they had completed questionnaires as part of the assessment process, yet they did not necessarily distinguish this experience, or this phase of the treatment, from the broader therapeutic course. Rather, they regarded it as a natural and integrated part of being in treatment. Assessments were not described as particularly helpful, nor as disruptive, they were viewed as an inherent and expected part of psychiatric care. This perception of assessments as routine may also reflect the patients’ prior experiences with mental health treatment, as both had undergone several previous treatment courses and were therefore familiar with the process of being assessed. From this perspective, the assessment did not appear as a distinct or novel element but rather as something expected and embedded within the broader framework of care. This may help explain why patients tended to speak about the assessment in general terms, without describing it as contributing directly to their understanding of illness, decision-making, or therapeutic progress.

Overall, the patients expressed neutral attitudes toward the PROMs. They did not recall the questionnaires as particularly challenging or as providing new insights. In Rami’s case, he remembered little from the questionnaires themselves, however, he mainly remembered the assessment as a way for the clinician to understand who he was as a person:

*“For me, I felt that the assessment process was very much about, you know, these questionnaires we had to fill in – it was about, you know, learning about me as a person.”*

This quote reflects a positive experience of the assessment process, where the patient feels listened to and recognized as an individual rather than merely as a “case”. While the quote does not indicate that this experience stemmed directly from completing the PROMs themselves, the broader analysis suggests that it is the clinician’s way of integrating and adapting the use of PROMs, along with their relational and communicative approach, that shapes this sense of being listened to and acknowledged. Thus, the relevance of PROMs is closely tied to how they are embedded within the therapeutic encounter and used to support relational understanding and dialogue.

##### 3.3.2.2 Relational embedding and shared use

This sense of integration also extended to the use of technology during the assessment process, which both patients and clinicians described as a natural part of the therapeutic setting rather than as something that disrupted it. Both clinicians emphasized how the computer screen could function as a *shared third*, as something that supported collaboration, transparency, and alliance in the therapeutic relationship. They both described physically positioning the screen so that both could see it, rather than having it turned solely toward the clinician. Robin emphasized that this setup fostered transparency and helped maintain dialogue during the session. Allowing both parties to view the screen made the process more interactive and relational, though it also required the clinician to be sufficiently familiar with the assessment tools to balance attention between the screen and the patient.

##### 3.3.2.3 From data collection to disuse

While the use of technology and patient assessment in general were regarded as natural elements of psychiatric care, both clinicians found the DTD battery to be overly comprehensive and, at times, excessive. In practice, it was not always feasible or meaningful to complete the full set of assessments for every patient, as the standardized format did not always align with individual needs and capacities. Furthermore, clinicians reported that DTD data were primarily used during the initial diagnostic and assessment phase and only rarely in the subsequent therapeutic process. In Robin’s case, the assessment data helped inform the overall diagnostic impression, but clinical judgment and information from prior patient records tended to carry greater weight in relation to treatment planning. Sam, in contrast, used assessment data for both diagnostic purposes and treatment planning, but noted that this occurred only during the initial phase:

*“And it’s been a long time since I had to do those [PROMs], so I don’t really think about them anymore. I’ve just forgotten them.”*

This quote illustrates how PROMs were primarily administered at baseline and played little or no role in the continuation of therapy, highlighting the gap between systematic data collection and its practical use in ongoing clinical work. This limited use of assessment data beyond the initial diagnostic phase illustrates how standardized tools, while designed to ensure systematic data collection, must constantly be negotiated and adapted in practice. This tension between standardized procedures and clinical flexibility is explored further in the final theme.

##### 3.3.2.4 Analytical summary

Assessments and PROMs thus occupy an ambiguous position: mundane yet relationally negotiated. Patients normalize them as routine, while clinicians humanize them through interactional adaptation. Their clinical influence lies less in numerical outputs than in how they are woven into the interpersonal dynamics of care.

#### 3.3.3 The tension between standardization and flexibility

Clinicians’ accounts highlight an ongoing negotiation between adhering to systematic procedures for data collection and adapting these to the realities of clinical practice. While standardized tools such as the International Trauma Questionnaire (ITQ) and the International Trauma Interview (ITI) are designed to promote consistency and comparability across cases, their overlap in content often leads to repetition and inefficiency. In both dyads, the clinicians described how the structured assessment workflow is continually adjusted, both at the individual and clinic level, to adapt to patients’ differing capacities for understanding and engagement. For example, patients with limited literacy may require that questions are explained or read aloud, transforming the self-report questionnaire into a dialogue resembling an interview. In such cases, the practical distinction between the ITQ and ITI becomes blurred to the clinicians. This overlap can create a sense of repetition for both patients and clinicians, as the same themes are addressed twice in different formats. To manage this, Robin explained that clinicians at the clinic have developed local solutions through supervision and shared reflection. One such adaptation involves skipping the ITQ if a patient is unable to complete it independently within approximately ten minutes and proceeding directly to the ITI. This adjustment reduces repetition while maintaining the overall purpose of both instruments. Sam also referred to this workflow during the interview.

Robin further described tailoring the assessment process to patients’ physical and psychological capacities. For example, patients experiencing chronic pain or significant distress may struggle to sit through a 90-minute assessment session. In such cases, the clinician prioritizes elements of the assessment battery to ensure that the process remains tolerable for the patient while retaining its clinical value. Similarly, Sam explained that his patient, Rami, suffered from back pain and required breaks during the assessment, which made it necessary to prioritize certain instruments. Sam generally emphasized making case-by-case judgments about which PROMs to prioritize and complete within the available time, noting that this process becomes easier with experience and familiarity with the assessment tools. These examples illustrate how standardized data collection protocols are interpreted and adapted into everyday practice.

##### 3.3.3.1 Analytical summary

Although assessments are applied systematically in principle, they are not treated as rigid procedures. Instead, they are continuously adapted to the needs and capacities of each individual patient, making the process both more manageable for patients and more meaningful for clinicians. While protocols seek comparability, clinicians act as mediators, translating standards into individually meaningful encounters. This flexibility underscores a central tension within assessment practice: balancing the demand for standardization and data quality with the realities of patient diversity, clinical judgment, and the need to maintain responsive and person-centered care, reflecting not only practical considerations but also ethical and relational commitments to patient wellbeing.

## 4 General discussion

This study combined system data, clinician survey responses, and clinician–patient dyadic interviews to provide a comprehensive understanding of how the PROM database was implemented and experienced in routine refugee mental health care. Across these three data sources, a coherent pattern emerged: while the idea of using PROMs to structure assessment and integrate the patient perspective was broadly supported, systematic use in practice remained limited. System data showed low completion rates beyond baseline, the survey data reflected practical and attitudinal barriers to routine use, and the dyadic interviews illuminated the perceived meaning of PROMs depended on their integration into the therapeutic relationship. Together, these findings point to a consistent tension between the promise of structured data collection and the realities of clinical work in complex care settings with difficult-to-treat disorders. This tension is reflected in a recent qualitative meta-analysis showing that patient-reported data in psychotherapy serves multiple roles beyond simple measurement: it provides shared markers, prompts reflection and emotional change, and can both strengthen and disrupt the therapeutic alliance while inviting strategic responses from patients (29). Framed this way, PROMs do not merely provide outcome measurement from the patient’s perspective; they actively participate in the therapeutic process shaping reflection, interaction, and alliance rather than simply recording them.

### 4.1 Rethinking implementation: from frameworks to co-creation

Although the PROM database was largely integrated into baseline assessment workflows, its use during treatment and follow-up was limited. The marked decline in completion rates beyond intake suggests difficulties in sustaining systematic monitoring across the care pathway. As around half of the patients were still active at the time of data retrieval, many had not yet reached end-of-treatment assessment, which partly explains the low follow-up completion rates. Studies from more standardized outpatient settings have shown that digital PROM systems can substantially increase completion when embedded within automated workflows and supported by feedback dashboards (30). In contrast, our results show that such gains did not occur in this high-complexity refugee context. This divergence points to contextual and organizational factors, such as unstable patient trajectories, interpreter-mediated communication, and varying clinician engagement, that likely constrained the potential “digital lift” in completion. This observation prompts reflection on our implementation approach. Although we aimed to avoid a top-down rollout, elements of our process still reflected conventional frameworks that presume relatively stable workflows and shared epistemic commitment. Both Wainberg et al. (31) and Olive et al. (30) argue for locally grounded, co-created implementation. Read together, these sources suggest that in our high-complexity context, characterized by interpreter-mediated care and unstable clinical trajectories, earlier, deeper, and more sustained co-creation involving clinicians, interpreters, and patients could have strengthened ownership and alignment, thereby improving completion rates and routine use.

Importantly, the present study did not aim to optimize PROM implementation per se, but to understand how clinicians and patients made sense of and found value in using PROMs within routine care. From this perspective, low completion rates are less an implementation failure than an indicator of the nuanced and context-dependent meanings attached to PROM use in everyday clinical practice. Clinicians appeared to use the PROM when it felt appropriate and relevant for the individual case, exercising clinical judgment rather than procedural compliance.

### 4.2 Beyond usability: the importance of epistemic fit

While previous studies highlight that intuitive and user-friendly technologies can improve PROM implementation, our findings suggest that usability alone cannot account for the low completion rates. The survey data or qualitative data did not indicate major complaints about the technical interface or accessibility of the system. Instead, barriers appeared to lie in more fundamental aspects, clinicians’ digital competence and, more importantly, their buy-in to the rationale for systematic PROM use. Evidence from prior implementation studies supports this interpretation: attitudes, expectations, and perceived clinical relevance among both clinicians and patients critically shape whether PROMs are meaningfully integrated into care (32). The dyadic interviews suggested that clinicians struggled to balance attention to the therapeutic alliance and the patient’s immediate needs with the structured administration of questionnaires intended to systematize patient perspectives for treatment planning. This tension may be particularly pronounced when symptom presentations are diffuse or somatically patterned, when avoidance limits the tolerability of symptom-focused questioning, or when co-occurring depressive reactions and ongoing stressors shape day-to-day functioning as is often the case in trauma-affected refugee populations (33, 34). In such contexts, clinicians may need clearer formulation-level bridges that connect PROM scores to shared clinical meaning and concrete next steps, rather than experiencing PROMs as an additional task that competes with alliance-building and immediate care priorities. This difficulty may also reflect a deeper epistemological tension between idiographic and nomothetic orientations in clinical work (35). Låver et al. (29) empirically map this epistemic friction: the same scores serve a nomothetic function for monitoring *and* an idiographic function by triggering self-awareness, reframing problems, or shifting the therapeutic narrative. Implementations that fail to accommodate the nuanced, idiographic potential of PROMs risk poor adoption, even when the system itself is technically sound. When PROMs are framed or experienced merely as instruments to reveal a patient’s “true” subjective health status, they offer little perceived relevance to the relational and interpretive work that defines clinical practice. In such cases, PROMs risk being viewed as administratively required rather than clinically valuable, whereas framing them as facilitators of meaning-making and therapeutic dialogue appears more congruent with clinicians’ professional priorities. From an implementation-science perspective, this suggests that sustainable PROM integration may depend less on technical optimization and more on epistemic alignment, how well the system’s underlying logic resonates with clinicians’ professional values and therapeutic frameworks. Implementation models that explicitly account for relational and contextual dimensions, beyond workflow fit and usability, may therefore be better suited to bridge the gap between data-driven standardization and the person-centered ethos of mental health care [see Wainberg et al. (31)].

### 4.3 Navigating vulnerability: balancing protection and participation

In the qualitative data, clinicians’ perceptions of patient vulnerability emerged as a key reason for adapting or omitting PROMs, warranting reflection on how such perceptions influence PROM implementation. We use the term in line with Luna (36) notion of layered, context-dependent exposure to risk, shaped by trauma, comorbidity, interpreter-mediated communication, and structural factors such as displacement, legal insecurity, and economic hardship. In this setting, clinicians often highlighted “vulnerability” to explain protective restraint, for example, by postponing or foregoing PROM administration to avoid overburdening patients. These protection dynamics are plausibly accentuated in refugee trauma care because presentations are frequently multimorbid and shaped by complex trauma sequelae (e.g., affect dysregulation, negative self-concept, relational disturbance) (37) alongside depression/anxiety (33, 38), sleep and somatic symptoms (39), and ongoing post-migration stressors (34, 40). In this context, PROM completion may be experienced as an additional demand that can evoke distress, shame, or overwhelm, particularly when it requires sustained attention to symptoms, trauma-related material, or functioning, thereby reinforcing clinicians’ inclination to defer or omit PROMs as a form of protection. While ethically motivated, this stance risks gatekeeping by excluding those whose perspectives PROMs are designed to include. Vulnerability should therefore prompt proportionate safeguards and adaptive procedures rather than exclusion. Plain-language and translated PROMs, interpreter-friendly formats, low-burden tools, flexible completion options, and consent understood as an ongoing dialogue can support inclusion. Framed in this way, PROMs may serve as relational supports that facilitate participation rather than administrative demands that strain it.

At the same time, the theme of vulnerability raises a deeper question: how can clinicians protect patients without silencing their participation? As argued by Roest et al. (41), vulnerability should not be seen as the absence of agency but as a condition that transforms how agency is expressed and negotiated in practice. In our study, clinicians’ protective restraint, postponing or moderating PROM use to avoid overburdening patients, reflected a strong ethical commitment to care. However, this protection-oriented stance could also have had unintended consequences, as it could leave patients with fewer opportunities to participate actively through PROMs. By limiting their use to situations judged “safe” or “appropriate”, clinicians inadvertently reduced patients’ chances to express their perspectives and engage in shared meaning-making - the very aims PROMs are intended to support. Supporting relational agency, where vulnerability and participation can coexist, requires expanding the ethical focus from control and harm-avoidance toward adaptation, dialogue, and mutual responsiveness. Ethical practice in such contexts may thus need to move beyond “protection from” toward “participation with,” enabling patients to engage safely yet meaningfully in their own care.

Reframing vulnerability as a relational signal for ethical adaptation (36, 42, 43) offers a constructive way forward. Rather than a fixed attribute, vulnerability can be understood as a situational condition that calls for flexible and proportionate engagement. From this view, protection and participation are not opposing aims but interdependent dimensions of ethical, person-centered care. Recognizing this interdependence may help clinicians and systems move from procedural inclusion toward genuine responsiveness where PROMs function as tools for dialogue and understanding rather than instruments of control.

### 4.4 Between care and evidence: negotiating the logics of implementation

The patterns described above extend beyond individual ethical reasoning and reveal structural tensions within the broader system of evidence-based mental health care. At stake is how clinicians navigate the demands of measurement and accountability while preserving the relational and contextual integrity of care. This flexible use of PROMs reflects patient-centered and ethically responsive practice, yet it also exposes a fundamental challenge within evidence-based care: aligning individualized, context-sensitive work with systems that privilege comparability, quantification, and procedural consistency (44). In other words, the very practices that sustain therapeutic integrity may sit uneasily within frameworks designed to secure standardization and accountability.

This challenge mirrors a deeper epistemic tension in PROM implementation - how to preserve the relational depth of therapeutic work while meeting system-level expectations for standardized data. Clinicians operate within overlapping institutional logics: the professional logic of person-centered care and the managerial logic of measurable evidence (45, 46). Decisions about when and how to use PROMs thus represent an ongoing negotiation between these value systems, as clinicians strive to protect the integrity of the therapeutic relationship while fulfilling organizational requirements for documentation and evaluation. In this sense, PROM use becomes a site where ethical, epistemic, and institutional commitments intersect and must be continually balanced.

### 4.5 Toward reflexive and rights-based implementation

Recognizing these intersecting logics invites a shift in how implementation itself is conceptualized. Rather than viewing the integration of PROMs as a matter of balancing competing demands, it may be more productive to see it as a process of mutual adaptation - where clinical judgment, patient experience, and institutional requirements inform one another over time. Building on this understanding, implementing PROM systems in complex clinical environments requires approaches that are both context-sensitive and reflexive.

Instead of treating ethical inclusion as a static principle, implementation must become an iterative process of learning in which patients, clinicians, and other relevant stakeholders co-shape how PROMs are used, interpreted, and valued. This means attending not only to usability and workflow but also to meaning, trust, and epistemic alignment as the fit between the system’s logic and the professional and cultural worlds in which it operates. Approaches grounded in co-design and participatory ethics (41, 43) can support this shift by embedding reflection and dialogue throughout the process. At a broader level, such methods exemplify rights-enabling practice: they safeguard patient participation without instrumentalizing it and strengthen, rather than strain, the relational foundations of mental health care.

### 4.6 Implications for future implementation research

Although our aim was not to optimize PROM implementation, the study design offers a transferable approach for examining how PROM systems are taken up, set aside, and made meaningful in routine care. Future implementation studies in complex trauma and refugee services could build on this learning-oriented model by triangulating (i) routine system-use indicators (e.g., completion and missingness across timepoints), (ii) clinician survey data capturing perceived usability and clinical value, and (iii) clinician-patient dyadic interviews examining how PROMs enter (or do not enter) the therapeutic relationship and care decisions. Further research along these lines would be strengthened by purposive sampling across high- and low-use clinicians and by treating interpreter-mediated delivery and local adaptations as core implementation data rather than contextual noise.

Building on our findings, subsequent work could examine the conditions under which PROMs achieve epistemic fit, i.e., when and how scores can be linked to shared formulation, therapeutic dialogue, and clinically relevant decision-making, alongside the ethical reasoning that underpins clinicians’ decisions to defer, adapt, or omit PROMs in situations of perceived vulnerability. Such work could evaluate implementation success on two levels: the uptake, coverage, and completeness required for nomothetic database purposes (including representativeness), and the extent to which PROM use supports clinical work (perceived value, communication and decision-making, and preservation of the patient perspective). To strengthen explanation of *how* and *why* these outcomes emerge, future studies could use realist-informed process evaluation (47), and incorporate complementary methods such as direct observation of PROM-related encounters (e.g., intake/discharge conferences and instances where PROMs are discussed in sessions), interviews with interpreters to capture how translation and interactional dynamics shape PROM use, and systematic documentation of local adaptations. Where the aim is to test strategies for improving uptake while monitoring clinical consequences, hybrid effectiveness–implementation designs offer a pragmatic next step (48).

## 5 Strengths and limitations

A key strength of this study lies in its multi-method design, which integrated system-level data, clinician survey responses, and clinician–patient dyadic interviews. This triangulation allowed for a rich understanding of both the measurable patterns of PROM use and the lived meanings attached to them in practice. By including perspectives from clinicians and patients within the same care encounters, the study provides unique insight into how PROMs are negotiated in relational, cross-cultural, and interpreter-mediated contexts which are settings that are rarely captured in implementation research. The combination of qualitative and quantitative data thus strengthens both the credibility and transferability of the findings.

However, several limitations should be noted. The study was conducted within a single specialized service context, which may limit generalizability to other clinical or cultural settings. The small sample of dyadic interviews, while providing depth, constrains the breadth of perspectives represented. As participation was voluntary, selection bias could also have influenced which clinicians and patients contributed to the qualitative material, potentially over-representing those more engaged or favorable toward PROM use. In addition, the study did not include direct observational data, which might have provided further insight into how PROMs are used or set aside in real-time clinical encounters.

Despite these limitations, the strength of the study lies in its ecological validity and conceptual contribution. By examining PROM implementation in a high-complexity, trauma-focused service, it highlights structural and epistemic tensions that are often overlooked in more standardized settings. The findings thus extend beyond a single context, offering broader lessons for evidence-based practice in mental health care that aspires to be both person-centered and ethically responsive.

## Data Availability

The raw data supporting the conclusions of this article will be made available by the authors, without undue reservation.
